# Why Can Insulin Resistance Be a Natural Consequence of Thyroid Dysfunction?

**DOI:** 10.4061/2011/152850

**Published:** 2011-09-19

**Authors:** Gabriela Brenta

**Affiliations:** Department of Endocrinology, Dr. César Milstein Hospital, La Rioja 951, C1221ACI, Buenos Aires, Argentina

## Abstract

Evidence for a relationship between T4 and T3 and glucose metabolism appeared over 100 years ago when the influence of thyroid hormone excess in the deterioration of glucose metabolism was first noticed. Since then, it has been known that hyperthyroidism is associated with insulin resistance. More recently, hypothyroidism has also been linked to decreased insulin sensitivity. The explanation to this apparent paradox may lie in the differential effects of thyroid hormones at the liver and peripheral tissues level. The purpose of this paper is to explore the effects of thyroid hormones in glucose metabolism and analyze the mechanisms whereby alterations of thyroid hormones lead to insulin resistance.

## 1. Introduction

The effects of T4 and T3 have a large impact on glucose homeostasis. This concept was acknowledged by Nobel Prize winner Dr. Bernardo Alberto Houssay in his lecture in 1947 “*The blood sugar and the production and consumption of glucose are kept within normal bounds, therefore there is an equilibrium between the glands of internal secretions which reduce the blood sugar (pancreas) and those which raise it (anterohypophysis, adrenals, thyroid, etc.)*”. Thyroid hormones exert both insulin agonistic and antagonistic actions in different organs. However, this occurs in a fine balance necessary for normal glucose metabolism. Deficit or excess of thyroid hormones can break this equilibrium leading to alterations of carbohydrate metabolism. Overt hyperthyroidism has been related to glucose intolerance and even ketoacidosis. With regards to hypothyroidism, cases of hypoglycemia have been reported in the literature despite the fact that peripheral insulin resistance may be present.

 In the century that has elapsed, since the first observations of uncontrolled glucose metabolism in thyrotoxic diabetic patients [1], new pathways involved in the regulation of glucose homeostasis by thyroid hormones have been unveiled. Novel findings include the stimulation of hepatic glucose production by thyroid hormones acting via a sympathetic pathway from the hypothalamus [2] and the discovery of transcriptional regulators of metabolic and mitochondrial genes that, influenced by intracellular T3 levels, may contribute to the development of insulin resistance [3]. The calorigenic-thermogenic activity of T3 long ascribed solely to uncoupling of mitochondrial oxidative phosphorylation has recently been related to T3-induced gating of mitochondrial permeability transition pore (PTP) of the inner mitochondrial membrane where the whole T3 transduction pathway integrates genomic and nongenomic activities of T3 in regulating mitochondrial energetics [4]. 

 In this paper, we summarize the effects of thyroid hormones in glucose metabolism and its alterations when thyroid dysfunction is present.

## 2. Effects of Thyroid Hormones on Glucose Metabolism (Figure 1)

### 2.1. Direct Effects of Thyroid Hormones at the Liver Level (Table 1)

Thyroid receptor-mediated effects on gene transcription and translation are key in the regulation of glucose metabolism. According to the results of studies with complementary DNA (cDNA) microarray analysis in mouse liver, this organ is a major target of thyroid hormones. Several genes involved in gluconeogenesis, glycogen metabolism, and insulin signaling that are regulated by thyroid hormones have been identified. In the study by Feng et al. [5], RNA from hypothyroid mice treated with T3 was prepared, labeled with fluorescent dye, and hybridized with the cDNA microarray. An increase in glucose-6-phosphatase mRNA expression with T3 was reported. This enzyme hydrolyzes glucose-6-phosphate and completes the final step in gluconeogenesis and glycogenolysis, therefore playing an important role in the homeostatic regulation of blood glucose levels. Another finding was a decrease in mRNA expression of Akt2 (protein kinase B), a serine/threonine kinase that is an essential molecule in the insulin signaling pathway. Akt2 has been shown to promote glycogen synthesis in liver by inactivating glycogen synthase kinase 3. Thus, a decrease in Akt2 activity would decrease glycogen synthesis explaining the antagonistic insulin effect of thyroid hormones at the liver. Moreover, an induction of *β*2-adrenergic receptor mRNA and repression of inhibitory G protein (Gi) RNA of the adenylate cyclase cascade by T3 were also reported. All these results are in favour of a permissive influence of T3 in the glycogenolytic and gluconeogenic effects of epinephrine and glucagon. Other hepatic gluconeogenic enzymes that have been found to be positively regulated by thyroid hormones include phosphoenolpyruvate carboxykinase (PEPCK), the enzyme that catalyzes the rate-controlling step of gluconeogenesis [6] and pyruvate carboxylase, involved in the synthesis of oxaloacetate from pyruvate [7]. The catalytic activity of pyruvate carboxylase has been found increased approximately 2-fold in hyperthyroid rats compared with untreated or treated euthyroid controls. 

 Another mechanism, whereby thyroid hormones are known to increase hepatic glucose output, is through increased hepatic expression of the glucose transporter GLUT2 [8] as previously shown in a rat model where GLUT2 protein concentration in crude liver membranes was twice as high in chronically hyperthyroid versus hypothyroid animals.

 It has been previously reported that, despite an expected resistance towards the insulin inhibitory effect on gluconeogenesis, the transcription of several enzymes involved in lipid synthesis or lipid metabolism is increased in hyperinsulinemic, insulin-resistant mice [9]. Furthermore, T3 induction of lipogenesis through the transcriptional activation of malic enzyme, involved in fatty acid synthesis, has been previously reported [10]. Therefore, it is possible that by the induction of lipogenic enzymes, T3 could be further aggravating the dysregulation of liver glucose and lipid metabolism characteristic of insulin resistance.

 As a result of the long time quest for thyroid analogs that possess the favourable actions on metabolism without the unwanted thyroid cardiac effects, an indirect way of learning about T3 action in the different tissues has emerged [11]. The differential distribution of thyroid receptor (TR) isoforms in the tissues has been key for the development of these analogs. With regards to lipogenesis, carbohydrate-response element-binding protein (ChREBP) is a major transcription factor controlling the activation of glucose-induced lipogenesis in liver and is a direct target of thyroid hormones in liver and white adipose tissue (WAT), the two main lipogenic tissues in mice. ChREBP is shown to be specifically regulated by TRbeta but not by TRalpha in vivo, in liver where TRbeta represents 80% of the thyroid hormone bound TR, but also in WAT where both TR isoforms are expressed [12]. Although the area of thyroid analogs is beyond the scope of this paper, it is to be mentioned that some thyromimetic analogs, such as 3,5-l-diiodothyronine (T(2)), exert their beneficial action on metabolism, without inducing a thyrotoxic state, through a mechanism that does not involve binding to thyroid hormone receptors [13]. In rats fed a high-fat diet, the addition of T(2) to lipid-overloaded cells resulted in reduction in lipid content; downregulation of peroxisome proliferator-activated receptors (PPAR)*α*, PPAR*γ*, and alternative oxidase (AOX) expression; increase in PPAR*δ* expression; and stimulation of mitochondrial uncoupling thus preventing and reversing hepatic steatosis in this animal model. Surprisingly, in this study, these lipid-lowering actions not mediated by TR were also observed with T3.

 To summarize, all these findings have helped to understand that thyroid hormones have insulin antagonistic effects at the liver that lead to an increased glucose hepatic output, via an enhanced rate of gluconeogenesis and glycogenolysis. With regards to lipid metabolism, both lipogenesis and lipolysis are stimulated by T3. However, in the context of insulin resistance, the conversion of glucose into fatty acids together with nonsuppressed gluconeogenesis is simply perpetuating the hyperinsulinemic state. Furthermore, nutritional influences, such as those of high-fat diets, should also be taken into consideration as modifiers of the effects of thyroid hormones on insulin sensitivity.

### 2.2. Direct Effects of Thyroid Hormones at the Peripheral Tissue Level (Table 1)

Opposite to what occurs at the liver level, at peripheral tissues, thyroid hormones have been shown to exert some of their actions synergically with insulin. The upregulation of the expression of genes such as GLUT-4 [14] or phosphoglycerate kinase (PGK) [15], involved in glucose transport and glycolysis respectively, is a good proof of concept.

In skeletal muscle, the main site of insulin-mediated glucose disposal, glucose transporter GLUT4, is induced by T3, revealing that it can increase basal and insulin-stimulated glucose transport in this tissue [14]. Another T3 target in skeletal muscle is mitochondrial uncoupling protein 3 (UCP3). Unveiling this association may be important since progressive reduction of UCP3 levels results in insulin resistance accompanied by decreased fatty acid oxidation and a less intense Akt/PKB and 5′ adenosine monophosphate-activated protein kinase (AMPK) signaling [16]. Although discrepancies between the regulation by T3 of UCP3 expression in rats, humans, and mice have been observed, the rat model has shed some light into T3 actions in this tissue. T3 intravenous (i.v.) administration in hypothyroid rats showed a rise in serum fatty acid levels concomitant with a rapid increase in UCP3 expression in gastrocnemius muscle. These findings point to UCP as a possible molecular determinant of the action of T3 on energy metabolism [17].

 Liver actions of the naturally occurring thyromimetic analog T2 have been discussed above. However, T2 actions have been also explored in skeletal muscle [18]. In a model of high-fat diet-induced insulin resistance in rat, the administration of T2, on the gastrocnemius muscle, induced remarkable changes on the metabolic/structural phenotype and insulin signaling. T2 increased insulin-stimulated Akt phosphorylation levels, the muscle contents of fast/glycolytic fibers and sarcolemmal GLUT4. Moreover, glycolytic enzymes and associated components were upregulated together with phosphofructokinase activity.

 The result of cDNA microarray analysis on skeletal muscle of a group of healthy men receiving 75 *μ*g/d of T3 for 14 days has shown that not only genes with agonistic insulin effects but several others with antagonistic insulin effects are upregulated by treatment with T3 [19], underlying the pleiotropic effect of thyroid hormone in energy metabolism. cDNA array data have also provided a molecular basis to the effect of T3 on adipose tissue. In human adipocytes, T3 increases the mRNA levels of the lipolytic *β*2-AR, favouring catecolamine-induced lipolysis and it also downregulates Sterol regulatory element binding protein (SREBP1c), involved in lipogenesis, which may constitute a link between hyperthyroidism and insulin resistance [20]. 

 Skin fibroblasts have been also used to study thyroid hormone-responsive genes involved in metabolism in human cells. Although they are not as metabolically active as hepatic cells, they are easily obtained and also, thyroid hormone-responsive. In cultured human fibroblasts, Moeller et al. [15] observed that, opposite to a posttranscriptional regulation as reported for other growth factors and hormones, the mRNA of the transcription factor HIF-1*α* (Hypoxia-inducible factor 1), a key mediator of glycolysis, increased in response to T3. As the glucose transporter GLUT1, several enzymes of glycolysis, and the lactate exporter SLC16A3 were all also found induced by T3 and are target genes of the transcription factor HIF-1*α*, the authors postulated that the effect of thyroid hormones on the induction of these genes most probably was indirect and HIF-1*α* mediated. Furthermore, a new mechanism of thyroid action was unraveled by this group of researchers [21]. It was shown that T3 bound to TRbeta, *in lieu* of initiating gene transcription in the nucleus, activates the phosphatidylinositol 3-kinase (PI3K) signaling pathway in the cytosol in order to activate HIF-1*α* gene expression.

 At the cellular level, thyroid hormones can also increase mitochondrial biogenesis, fatty acid oxidation, and TCA cycle activity [22]. These findings are quite relevant since the role of mitochondrial dysfunction, leading to cellular lipid excess and impaired oxidative metabolism, has been clearly demonstrated in the pathogenesis of type 2 diabetes [23–25]. Furthermore, it has been described that in skeletal muscle, the lack of thyroid hormones might dysregulate mitochondrial gene expression [26]. PPAR gamma coactivator-1 alpha (PGC-1 alpha), a key transcriptional regulator of mitochondrial content and function, fatty acid oxidation, and gluconeogenesis, has been involved in the process whereby thyroid hormones regulate mitochondrial function [3]. It has been shown that PGC-1 alpha gene expression is increased by T3, as much as 13-fold 6 hours after T3 treatment [27]. The regulation pattern of T3 on PGC-1 alpha is complex and may occur through nongenomic activation of kinases to induce the expression of PGC-1 alpha or through transcriptional upregulation via the presence of a thyroid responsive element (TRE) in the PGC-1 alpha promoter or by genomic upregulation of a transcription factor (via a TRE), which then binds to the PGC-1 alpha promoter and increases PGC-1 alpha transcription [28]. It is hypothesized that PGC-1 alpha can be dysregulated by reduced T3 levels [3], thus contributing to insulin resistance. Not only low circulating but also, intracellular T3 levels, could count for this matter. A lower expression and activity of type 2 iodothyronine-deiodinase (D2), the enzyme that is key for the conversion of T4 into T3 in muscle and thus, amplifies thyroid hormone signaling in individual cells, has been found linked to insulin resistance [29, 30]. If PGC-1 alpha effects on mitochondrial gene expression may indeed be regulated by thyroid hormone, normal activity of deiodinase type 2 is very relevant. Several factors, related to this enzymatic activity, are being currently studied. Bile acids are potent stimulators of the enzyme and may play an important role in the relationship between thyroid action and glucose metabolism [31]. On the other hand, the natural occurrence of polymorphisms of deiodinase type 2 such as Thr92Ala, with a lower activity, has also been implicated with increased risk for diabetes type 2 [29].

### 2.3. Indirect Effects of Thyroid Hormones to the Liver

It has been shown that the hypothalamus can modulate endogenous glucose production by using functionally reciprocal sympathetic and parasympathetic autonomic outputs to the liver [32]. Moreover, a sympathetic pathway from the hypothalamic paraventricular nucleus to the liver has been proposed as a central pathway for modulation of hepatic glucose metabolism by thyroid hormone [33]. Klieveric et al. [33] demonstrated that upon selective administration to the paraventricular nucleus (PVN), T3 increases endogenous glucose production and plasma glucose, and that these hypothalamic T3 effects are mediated via sympathetic projections to the liver. In order to arrive to such remarkable findings, the authors worked with euthyroid rats treated with methimazole and T4. First they administered an intracerebroventricular (i.c.v.) T3 or vehicle (Veh) infusion, and there was a significant increase in plasma glucose compared with Veh-treated rats. This meant that central T3 infusion could reproduce the characteristic increase in hepatic glucose output of thyrotoxicosis. To further identify the neuroanatomic region responsible for these changes, the authors infused T3 within the hypothalamus at the PVN. A similar response was obtained, that was independent of plasma T3, insulin, and corticosterone concentrations. They repeated the experiment in surgically hepatic sympathectomized animals (HSx) and sham-denervated animals. HSx animals showed a decrease of endogenous glucose output upon hypothalamic T3 infusion. The principal finding of this study is the description of a neural (autonomic) modulation of hepatic glucose metabolism by T3 at the hypothalamus that takes place independently of plasma glucoregulatory hormone concentrations.

## 3. Insulin Resistance as a Consequence of Hyperthyroidism

Thyrotoxic subjects frequently show impaired glucose tolerance. This is a result of increased glucose turnover with increased glucose absorption through the gastrointestinal tract, postabsorptive hyperglycemia, elevated hepatic glucose output, with elevated fasting and/or postprandial insulin and proinsulin levels, elevated free fatty acid concentrations and elevated peripheral glucose transport and utilization. The literature about this topic is vast and has been previously comprehensively reviewed by Dimitriadis and Raptis [34]. Thyrotoxic diabetic patients are more prone to ketosis [35]. Although ketoacidosis may result *per se* from the insulin resistance present in thyrotoxicosis, the stimulatory action of thyroid hormones in excess on lipolysis and free fatty acids availability can also contribute to increased ketogenesis [36].

### 3.1. Increased Hepatic Glucose Output in Hyperthyroidism

Thyrotoxicosis has been reported to increase endogenous glucose production in the liver in the basal state and to decrease hepatic insulin sensitivity in humans [37]. The different mechanisms to explain this phenomenon include increased rates of gluconeogenesis and glycogenolysis [38] mainly explained by the above-mentioned effects on the liver by thyroid hormones. To summarize, these effects include thyroid receptor-mediated effects on liver gene transcription [5], increased sympathetic action in the liver mediated by hypothalamus [33], and increased concentrations of the GLUT2 glucose transporters in the liver plasma membrane that allows for glucose efflux [8, 39] together with increased concentration of free fatty acids in plasma [40].

### 3.2. Peripheral Tissues Glucose Metabolism in Hyperthyroidism

The interpretation of the effects of hyperthyroidism on glucose utilization by peripheral tissues is by far the most complex issue on this topic. On one hand, the rates of glucose uptake in peripheral tissues have been found increased by thyroid hormones, suggesting that glucose utilization is highly increased, specially in skeletal muscle [34, 37, 41–44]. This increased utilization, as shown by indirect calorimetry during euglycemic hyperinsulinemic clamps, is mainly due to an increase in insulin-stimulated glucose oxidation rates [43, 45–48]. However, a decrease in insulin-stimulated nonoxidative glucose disposal, through reduced glycogenogenesis [43, 44, 49], takes place, with intracellular glucose being redirected towards glycolysis and lactate formation. The release of lactate from peripheral tissues back to the liver is a major contributor to the Cori cycle where more hepatic glucose is being produced [43, 49–51].

 Although glucose intolerance in hyperthyroidism can be easily explained by hepatic insulin resistance without involvement of peripheral tissues, impaired insulin-stimulated peripheral glucose uptake has also been proven in some studies. By means of the arteriovenous difference technique in the forearm muscles of hyperthyroid subjects after the consumption of a mixed meal, it has been clearly demonstrated that muscle blood flow is increased, masking a defect in insulin-stimulated glucose uptake [52]. Moreover, in disagreement with previous reports [41, 45], Shen et al. [53] also described decreased peripheral insulin sensitivity in hyperthyroidism.

Alternative explanations for peripheral insulin resistance in hyperthyroidism include an increased secretion of bioactive mediators (adipokines) such as interleukin 6 (IL6) and tumour necrosis factor a (TNF*α*) from adipose tissue in hyperthyroidism [54]. These adipokines, that exert both proinflammatory and insulin resistant effects, have been found elevated in hyperthyroid women [54].

### 3.3. Insulin and Glucagon Secretion and Degradation in Hyperthyroidism

In hyperthyroidism, decreased, normal, or even increased levels of plasma insulin have been reported [34]. However, a rather consistent finding has been the increased degradation of insulin in hyperthyroid subjects [43, 55]. It has been postulated that, in the long run, severe thyrotoxicosis can lead to irreversible pancreatic damage [56, 57]. 

With regards to glucagon, its secretion and metabolic clearance rates have been reported increased, explaining the normal fasting plasma levels described in hyperthyroidism [58].

### 3.4. Subclinical Hyperthyroidism and Insulin Resistance

Subclinical hyperthyroidism has also been associated with insulin resistance [59–61] in some but not all studies [62]. The heterogenous nature of this condition can partly explain this controversy. Endogenous subclinical hyperthyroidism may have a larger impact on glucose metabolism due to its chronicity and higher T3 levels when compared to exogenous administration of T4 [61].

## 4. Hypothyroidism Can Also Lead to Insulin Resistance

Although seldom happening, hypothyroid patients can experience hypoglycaemia. This phenomenon can be interpreted in the light of reduced levels of gluconeogenesis leading to decreased liver glucose output [63, 64]. On the other hand, insulin resistance has been shown to be present in peripheral tissues in hypothyroid animal models [65, 66]. Hence, a poor utilization of glucose in hypothyroidism may be offset by a reduced release to circulation maintaining a balance of the glucose metabolism. 

### 4.1. Animal Studies Showing Insulin Resistance in Hypothyroidism

Studies performed in adipocytes and skeletal muscle of rats made hypothyroid have shown that these tissues are less responsive to insulin with regards to glucose metabolism [63, 65–69]. Czech et al. [65] studied insulin responsiveness in adipocytes and skeletal muscle of mature rats rendered hypothyroid by a low iodine diet and propylthiouracil. It was observed that glucose conversion to glycogen was partially inhibited while the glycolytic flux stimulation by insulin was totally frustrated. This decrease in insulin sensitivity occurred without an impaired membrane insulin effector system. Other authors [66, 67] showed decreased insulin-stimulated glucose transport and/or phosphorylation, as well as a lower rate of glycolysis in the isolated, incubated soleus muscle of the hypothyroid rat and also suggested that the effects of hypothyroidism in muscle were not mediated through an interference of insulin binding to its receptor. It was postulated that they rather occurred through a postreceptor mechanism that may include abnormal phosphorylation of insulin signaling proteins.

Insulin resistance was confirmed in another study of rats with mild hypothyroidism [69]. In this study, insulin responsiveness was measured by an insulin tolerance test and an euglycemic-hyperinsulinemic clamp followed by measurements of tissue-specific glucose utilization indices with the labelled 2-deoxy-D-[1-3H]glucose (2-DG) technique in muscles (red quadriceps) and white adipose tissue (epididymal fat). Several other parameters were also determined such as muscle triglyceride content, plasma leptin and nonesterified fatty acids (NEFA) levels, and mRNA expression of resistin in adipose tissue as well as adipose tissue and liver carnitine palmitoyl transferase 1*α* (CPT-1*α*), and muscle carnitine palmitoyl transferase 1*β* (CPT-1*β*) mRNA levels, explored by real-time quantitative PCR. Plasma leptin levels were lower and adipose tissue mRNA expression of resistin higher, in the hypothyroid state. Looking for a potential role of leptin in the metabolic consequences of hypothyroidism, leptin was infused, and it was found that glucose disposal was recovered. Increased expression of muscle and adipose tissue carnitine palmitoyl transferases, decreased plasma NEFA levels, and reduced muscle triglyceride content after leptin infusion were interpreted as the mediators of the recovery of the insulin resistant state. Therefore, one possible explanation to decreased insulin responsiveness in hypothyroidism, according to the authors of this study, includes a dysregulation of leptin action at the hypothalamus. 

 Adipocyte-myocyte crosstalk by adipokines has been reported to play a significant role in skeletal muscle insulin resistance and may partially explain insulin resistance present in hypothyroidism [70]. However, other factors associated with insulin resistance in hypothyroidism, such as altered blood flow, impaired GLUT4 translocation, decreased glycogen synthesis, and decreased muscle oxidative capacity have to be also considered [71].

### 4.2. Studies in Humans Demonstrating Insulin Resistance in Hypothyroidism

Compared to the number of reports about insulin resistance in hyperthyroid patients, there are relatively fewer studies in humans dealing with the effects of hypothyroidism on glucose metabolism. Rochon et al. [72] measured whole-body sensitivity of glucose disposal to insulin in hypothyroid patients using the euglycemic-hyperinsulinemic clamp technique. They demonstrated that hypothyroidism induced a decrease in the insulin-mediated glucose disposal that reverted upon treatment. Similar results were obtained by Stanická et al. [73]. By means of the same clamp technique and also measuring glucose tolerance and beta-cell activity with an oral glucose tolerance tests (OGTT), Handisurya et al. [74] confirmed these findings and added the knowledge that glucose-induced insulin secretion is diminished by thyroid replacement corresponding well with the observed improvement of insulin sensitivity.

Dimitriadis et al. [75] explored glucose uptake in muscle and adipose tissue of hypothyroid and control subjects by means of the arteriovenous difference technique in the anterior abdominal subcutaneous adipose tissue and the forearm muscles after the consumption of a mixed meal. A decreased net extraction of glucose and blood flow after the meal in hypothyroid muscle and adipose tissue was reported. This impairment in the ability of insulin to increase blood flow rate to the hypothyroid tissues is an alternative explanation to the mechanism whereby hypothyroidism can induce lower glucose disposal. The short i.v. insulin tolerance test has been used to explore insulin sensitivity in acute overt hypothyroid patients. Compared to euthyroid controls, hypothyroid patients had a significantly lower glucose disposal [76].

Some negative results, however, in this field have been reported. A previous study in overt hypothyroid patients based on the homeostasis model assessment (HOMA-IR) [77] showed no association between hypothyroidism and insulin sensitivity. Moreover, Harris et al. [78] found unimpaired insulin-stimulated glucose disposal in the forearm of hypothyroid patients after treatment with levothyroxine.

### 4.3. Subclinical Hypothyroidism

With regards to subclinical hypothyroidism, insulin resistance has been demonstrated in some [60, 74, 79] but not all studies, where HOMA levels were found comparable to a control group [80–82]. However, in some of these negative studies [80, 81], hyperinsulinemia was reported in subclinical hypothyroid subjects and interpreted as an early sign of impairment of glucose metabolism.

## 5. Conclusions

Thyroid hormones have a large impact on glucose metabolism. A direct regulation on thyroid responsive genes at the target organ has been described and more recently an indirect effect involving hypothalamic pathways that regulate glucose metabolism via control of the sympathetic nervous system has been reported. Furthermore, thyroid hormone effects can be insulin agonistic, such as demonstrated in muscle or antagonistic such as observed in the liver. In hyperthyroidism, dysregulation of this balance may end in glucose intolerance mainly due to hepatic insulin resistance. In hypothyroidism the results are less evident. However, the available data suggest that insulin resistance is present mainly at the peripheral tissues. Possible explanations hypothesized to explain this phenomenon span from the dysregulation of mitochondrial oxidative metabolism to the reduction of blood flow in muscle and adipose tissue under hypothyroid conditions.

## Figures and Tables

**Figure 1:**
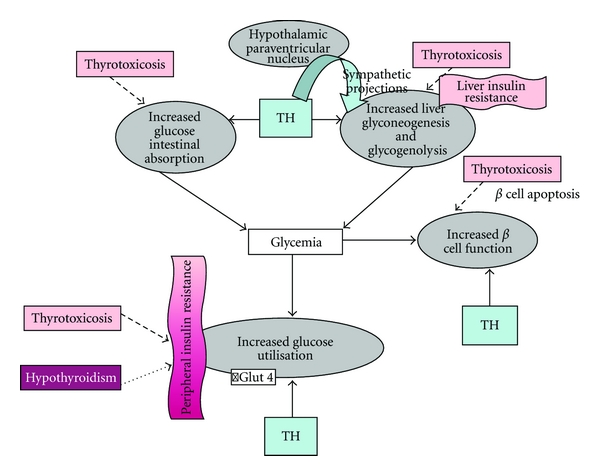
Effects of thyroid hormones on glucose metabolism in euthyroid (solid lines), hyperthyroid (rough-dashed lines), and hypothyroid conditions (fine-dashed lines). TH: thyroid hormones.

**Table 1:** Direct effects of T3 on genes that regulate glucose homeostasis at the liver and peripheral tissues (muscle, fat tissue, and fibroblasts).

Gene	Expression	Site	Net effect
glucose-6-phosphatase [5]	Increase	liver	Increase gluconeogenesis and glycogenolysis
protein kinase B (Akt2) [5]	decrease	liver	Decrease glycogen synthesis
*β*2-adrenergic receptor [5]	Increase	liver	Increase gluconeogenesis and glycogenolysis
inhibitory G protein (Gi) [5]	decrease	liver	Increase gluconeogenesis and glycogenolysis
phosphoenolpyruvate carboxykinase (PEPCK) [6]	Increase	liver	Increase gluconeogenesis
pyruvate carboxylase (PC) [7]	Increase	liver	Increase gluconeogenesis
GLUT2 [8]	Increase	liver	Increase glucose output
malic enzyme [10]	Increase	liver	lipogenesis
Carbohydrate-response element-binding protein (ChREBP) [12]	Increase	liver and fat tissue	lipogenesis
GLUT1 [14]	Increase	peripheral tissues	Increase glucose transport (basal)
GLUT4 [14]	Increase	peripheral tissues	Increase glucose transport (insulin-induced)
*β*2-adrenergic receptor [20]	Increase	Peripheral tissues	Increase lipolysis
phosphoglycerate kinase (PGK) [15]	Increase	peripheral tissues	Increase glycolysis
Hypoxia-inducible factor 1 (HIF-1*α*) [15]	Increase	peripheral tissues	Increase glycolysis
PPAR gamma coactivator-1 alpha (PGC-1 alpha) [27]	Increase	peripheral tissues	Increase mitochondrial biogenesis and function
uncoupling protein 3 (UCP3) [17]	Increase	peripheral tissues	Increase mitochondrial energy expenditure

## References

[1] Rohdenburg GL (1920). Thyroid diabetes. *Endocrinology*.

[2] Klieverik LP, Janssen SF, Van Riel A (2009). Thyroid hormone modulates glucose production via a sympathetic pathway from the hypothalamic paraventricular nucleus to the liver. *Proceedings of the National Academy of Sciences of the United States of America*.

[3] Crunkhorn S, Patti ME (2008). Links between thyroid hormone action, oxidative metabolism, and diabetes risk?. *Thyroid*.

[4] Yehuda-Shnaidman E, Kalderon B, Azazmeh N, Bar-Tana J (2010). Gating of the mitochondrial permeability transition pore by thyroid hormone. *Federation of American Societies for Experimental Biology Journal*.

[5] Feng X, Jiang Y, Meltzer P, Yen PM (2000). Thyroid hormone regulation of hepatic genes in vivo detected by complementary DNA microarray. *Molecular Endocrinology*.

[6] Park EA, Jerden DC, Bahouth SW (1995). Regulation of phosphoenolpyruvate carboxykinase gene transcription by thyroid hormone involves two distinct binding sites in the promoter. *Biochemical Journal*.

[7] Weinberg MB, Utter MF (1979). Effect of thyroid hormone on the turnover of rat liver pyruvate carboxylase and pyruvate dehydrogenase. *Journal of Biological Chemistry*.

[8] Weinstein SP, O’Boyle E, Fisher M, Haber RS (1994). Regulation of GLUT2 glucose transporter expression in liver by thyroid hormone: evidence for hormonal regulation of the hepatic glucose transport system. *Endocrinology*.

[9] Becker W, Kluge R, Kantner T (2004). Differential hepatic gene expression in a polygenic mouse model with insulin resistance and hyperglycemia: evidence for a combined transcriptional dysregulation of gluconeogenesis and fatty acid synthesis. *Journal of Molecular Endocrinology*.

[10] Mariash CN, McSwigan CR, Towle HC, Schwartz HL, Oppenheimer JH (1981). Glucose and triiodothyronine both induce malic enzyme in the rat hepatocyte culture: evidence that triiodothyronine multiplies a primary glucose-generated signal. *Journal of Clinical Investigation*.

[11] Brenta G, Danzi S, Klein I (2007). Potential therapeutic applications of thyroid hormone analogs. *Nature Clinical Practice Endocrinology & Metabolism*.

[12] Gauthier K, Billon C, Bissler M (2010). Thyroid hormone receptor *β*(TR*β*) and liver X receptor (LXR) regulate carbohydrate-response element-binding protein (ChREBP) expression in a tissue-selective manner. *Journal of Biological Chemistry*.

[13] Grasselli E, Voci A, Canesi L (2011). Non-receptor-mediated actions are responsible for the lipid-lowering effects of iodothyronines in FaO rat hepatoma cells. *Journal of Endocrinology*.

[14] Weinstein SP, O’Boyle E, Haber RS (1994). Thyroid hormone increases basal and insulin-stimulated glucose transport in skeletal muscle. The role of GLUT4 glucose transporter expression. *Diabetes*.

[15] Moeller LC, Dumitrescu AM, Walker RL, Meltzer PS, Refetoff S (2005). Thyroid hormone responsive genes in cultured human fibroblasts. *Journal of Clinical Endocrinology & Metabolism*.

[16] Senese R, Valli V, Moreno M (2010). Uncoupling protein 3 expression levels influence insulin sensitivity, fatty acid oxidation, and related signaling pathways. *Pflugers Archiv European Journal of Physiology*.

[17] de Lange P, Feola A, Ragni M (2007). Differential 3,5,3′-triiodothyronine-mediated regulation of uncoupling protein 3 transcription: role of fatty acids. *Endocrinology*.

[18] Moreno M, Silvestri E, De Matteis R 3,5-Diiodo-L-thyronine prevents high-fat-diet-induced insulin resistance in rat skeletal muscle through metabolic and structural adaptations.

[19] Clément K, Viguerie N, Diehn M (2002). In vivo regulation of human skeletal muscle gene expression by thyroid hormone. *Genome Research*.

[20] Viguerie N, Millet L, Avizou S, Vidal H, Larrouy D, Langin D (2002). Regulation of human adipocyte gene expression by thyroid hormone. *Journal of Clinical Endocrinology & Metabolism*.

[21] Moeller LC, Cao X, Dumitrescu AM, Seo H, Refetoff S (2006). Thyroid hormone mediated changes in gene expression can be initiated by cytosolic action of the thyroid hormone receptor beta through the phosphatidylinositol 3-kinase pathway. *Nuclear Receptor Signaling*.

[22] Goglia F, Moreno M, Lanni A (1999). Action of thyroid hormones at the cellular level: the mitochondrial target. *Federation of the Societies of Biochemistry and Molecular Biology Letters*.

[23] Patti ME, Butte AJ, Crunkhorn S (2003). Coordinated reduction of genes of oxidative metabolism in humans with insulin resistance and diabetes: potential role of PGC1 and NRF1. *Proceedings of the National Academy of Sciences of the United States of America*.

[24] Mootha VK, Lindgren CM, Eriksson KF (2003). PGC-1*α*-responsive genes involved in oxidative phosphorylation are coordinately downregulated in human diabetes. *Nature Genetics*.

[25] Petersen KF, Befroy D, Dufour S (2003). Mitochondrial dysfunction in the elderly: possible role in insulin resistance. *Science*.

[26] Irrcher I, Adhihetty PJ, Sheehan T, Joseph AM, Hood DA (2003). PPAR*γ* coactivator-1*α* expression during thyroid hormone- and contractile activity-induced mitochondrial adaptations. *American Journal of Physiology—Cell Physiology*.

[27] Weitzel JM, Radtke C, Seitz HJ (2001). Two thyroid hormone-mediated gene expression patterns in vivo identified by cDNA expression arrays in rat. *Nucleic Acids Research*.

[28] Irrcher I, Walkinshaw DR, Sheehan TE, Hood DA (2008). Thyroid hormone (T3) rapidly activates p38 and AMPK in skeletal muscle in vivo. *Journal of Applied Physiology*.

[29] Mentuccia D, Proietti-Pannunzi L, Tanner K (2002). Association between a novel variant of the human type 2 deiodinase gene Thr92Ala and insulin resistance: evidence of interaction with the Trp64Arg variant of the *β*-3-adrenergic receptor. *Diabetes*.

[30] Dora JM, Machado WE, Rheinheimer J, Crispim D, Maia AL (2010). Association of the type 2 deiodinase Thr92Ala polymorphism with type 2 diabetes: case-control study and meta-analysis. *European Journal of Endocrinology*.

[31] Watanabe M, Houten SM, Mataki C (2006). Bile acids induce energy expenditure by promoting intracellular thyroid hormone activation. *Nature*.

[32] Kalsbeek A, La Fleur S, Van Heijningen C, Buijs RM (2004). Suprachiasmatic GABAergic inputs to the paraventricular nucleus control plasma glucose concentrations in the rat via sympathetic innervation of the liver. *Journal of Neuroscience*.

[33] Klieverik LP, Janssen SF, Van Riel A (2009). Thyroid hormone modulates glucose production via a sympathetic pathway from the hypothalamic paraventricular nucleus to the liver. *Proceedings of the National Academy of Sciences of the United States of America*.

[34] Dimitriadis GD, Raptis SA (2001). Thyroid hormone excess and glucose intolerance. *Experimental and Clinical Endocrinology and Diabetes*.

[35] Beylot M, Riou JP, Bienvenu F, Mornex R (1980). Increased ketonaemia in hyperthyroidism. *Diabetologia*.

[36] Beylot M (1996). Regulation of in vivo ketogenesis: role of free fatty acids and control by epinephrine, thyroid hormones, insulin and glucagon. *Diabetes and Metabolism*.

[37] Cavallo-Perin P, Bruno A, Boine L, Cassader M, Lenti G, Pagano G (1988). Insulin resistance in Graves’ disease: a quantitative in-vivo evaluation. *European Journal of Clinical Investigation*.

[38] Sestoft L, Bartels PD, Flero P, Folke M, Gammeltoft S, Kristensen LO (1977). Influence of thyroid state on the effects of glycerol on gluconeogenesis and energy metabolism in perfused rat liver. *Biochimica et Biophysica Acta*.

[39] Mokuno T, Uchimura K, Hayashi R (1999). Glucose transporter 2 concentrations in hyper- and hypothyroid rat livers. *Journal of Endocrinology*.

[40] Saunders J, Hall SEH, Sonksen PH (1980). Glucose and free fatty acid turnover in thyrotoxicosis and hypothyroidism, before and after treatment. *Clinical Endocrinology*.

[41] Laville M, Rio JP, Bougneres PF, Mornex R (1984). Glucose metabolism in experimental hyperthyroidism: intact in vivo sensitivity to insulin with abnormal binding and increased glucose turnover. *Journal of Clinical Endocrinology & Metabolism*.

[42] Bratusch-Marrain PR, Gasić S, Waldhäusl WK (1984). Triiodothyronine increases splanchnic release and peripheral uptake of glucose in healthy humans. *The American Journal of Physiology*.

[43] Dimitriadis G, Baker B, Marsh H (1985). Effect of thyroid hormone excess on action, secretion, and metabolism of insulin in humans. *The American Journal of physiology*.

[44] Randin J, Scarriga B, Jequier F, Felber J (1985). Studies of glucose and lipid metabolism and continues indirect calorimetry in Graves’ disease: effect of an oral glucose load. *Journal of Clinical Endocrinology & Metabolism*.

[45] McCulloch AJ, Nosadini R, Pernet A (1983). Glucose turnover and indices of recycling in thyrotoxicosis and primary thyroid failure. *Clinical Science*.

[46] Sandler MP, Robinson RP, Rabin D, Lacy WW, Abumrad NN (1983). The effect of thyroid hormones on gluconeogenesis and forearm metabolism in man. *Clinical Endocrinology & Metabolism*.

[47] Celsing F, Blomstrand E, Melichna J (1986). Effect of hyperthyroidism on fibre-type composition, fibre area, glycogen content and enzyme activity in human skeletal muscle. *Clinical Physiology*.

[48] Foss MC, Paccola GMGF, Saad MJA, Pimenta WP, Piccinato CE, Iazigi N (1990). Peripheral glucose metabolism in human hyperthyroidism. *Journal of Clinical Endocrinology & Metabolism*.

[49] Dimitriadis GD, Leighton B, Vlachonikolis IG (1988). Effects of hyperthyroidism on the sensitivity of glycolysis and glycogen synthesis to insulin in the soleus muscle of the rat. *Biochemical Journal*.

[50] Parry-Billings M, Dimitriadis GD, Leighton B (1990). Effects of hyperthyroidism and hypothyroidism on glutamine metabolism by skeletal muscle of the rat. *Biochemical Journal*.

[51] Leighton B, Dimitriadis GD, Oarry-Billings M, Bond J, Kemp P, Newsholme EA (1990). Thyroid hormone analogue SKF L-94901: effects on amino acid and carbohydrate metabolism in rat skeletal muscle in vitro. *Biochemical Pharmacology*.

[52] Dimitriadis G, Mitrou P, Lambadiari V (2008). Insulin-stimulated rates of glucose uptake in muscle in hyperthyroidism: the importance of blood flow. *Journal of Clinical Endocrinology & Metabolism*.

[53] Shen DC, Davidson MB, Kuo SW, Sheu WH (1988). Peripheral and hepatic insulin antagonism in hyperthyroidism. *Journal of Clinical Endocrinology & Metabolism*.

[54] Mitrou P, Boutati E, Lambadiari V (2010). Insulin resistance in hyperthyroidism: the role of IL6 and TNF*α*. *European Journal of Endocrinology*.

[55] Randin JP, Tappy L, Scazziga B (1986). Insulin sensitivity and exogenous insulin clearance in Graves’ disease. Measurement by the glucose clamp technique and continuous indirect calorimetry. *Diabetes*.

[56] Lenzen S, Kucking H (1982). Inhibition of insulin secretion by L-thyroxine and thyroxine treatment in rats under the influence of drugs affecting the adrenergic nervous system. *Acta Endocrinologica*.

[57] Ximenes HM, Lortz S, Jörns A, Lenzen S (2007). Triiodothyronine (T3)-mediated toxicity and induction of apoptosis in insulin-producing INS-1 cells. *Life Sciences*.

[58] Dimitriadis G, Hatziagelaki E, Mitrou P (2011). Effect of hyperthyroidism on clearance and secretion of glucagon in man. *Experimental and Clinical Endocrinology & Diabetes*.

[59] Yavuz DG, Yüksel M, Deyneli O, Ozen Y, Aydin H, Akalin S (2004). Association of serum paraoxonase activity with insulin sensitivity and oxidative stress in hyperthyroid and TSH-suppressed nodular goitre patients. *Clinical Endocrinology*.

[60] Maratou E, Hadjidakis DJ, Peppa M (2010). Studies of insulin resistance in patients with clinical and subclinical hyperthyroidism. *European Journal of Endocrinology*.

[61] Rezzonico J, Niepomniszcze H, Rezzonico M, Pusiol E, Alberto M, Brenta G (2011). The association of insulin resistance with subclinical thyrotoxicosis. *Thyroid*.

[62] Heemstra KA, Smit JW, Eustatia-Rutten CF (2006). Glucose tolerance and lipid profile in longterm exogenous subclinical hyperthyroidism and the effects of restoration of euthyroidism, a randomised controlled trial. *Clinical Endocrinology*.

[63] McCulloch AJ, Johnston DG, Baylis PH (1983). Evidence that thyroid hormones regulate gluconeogenesis from glycerol in man. *Clinical Endocrinology*.

[64] Okajima F, Ui M (1979). Metabolism of glucose in hyper and hypothyroid rats in vivo. Glucose turnover values and futile cycle activities obtained with 14 C and 3H labelled glucose. *Biochemical Journal*.

[65] Czech MP, Malbon CC, Kerman K, Gitomer W, Pilch PF (1980). Effect of thyroid status on insulin action in rat adipocytes and skeletal muscle. *Journal of Clinical Investigation*.

[66] Dimitriadis GD, Leighton B, Parry-Billings M, West D, Newsholme EA (1989). Effects of hypothyroidism on the sensitivity of glycolysis and glycogen synthesis to insulin in the soleus muscle of the rat. *Biochemical Journal*.

[67] Dimitriadis G, Parry-Billings M, Bevan S (1997). The effects of insulin on transport and metabolism of glucose in skeletal muscle from hypothyroid rats. *European Journal of Clinical Investigation*.

[68] Dubaniewicz A, Kaciuba-Uscilko H, Nazar K, Budohoski L (1989). Sensitivity of the soleus muscle to insulin in resting and exercising rats with experimental hypo- and hyper-thyroidism. *Biochemical Journal*.

[69] Cettour-Rose P, Theander-Carrillo C, Asensio C (2005). Hypothyroidism in rats decreases peripheral glucose utilization, a defect partially corrected by central leptin infusion. *Diabetologia*.

[70] Havekes B, Sauerwein HP (2010). Adipocyte-myocyte crosstalk in skeletal muscle insulin resistance; is there a role for thyroid hormone?. *Current Opinion in Clinical Nutrition and Metabolic Care*.

[71] Peppa M, Koliaki C, Nikolopoulos P, Raptis SA (2010). Skeletal muscle insulin resistance in endocrine disease. *Journal of Biomedicine and Biotechnology*.

[72] Rochon C, Tauveron I, Dejax C (2003). Response of glucose disposal to hyperinsulinaemia in human hypothyroidism and hyperthyroidism. *Clinical Science*.

[73] Stanická S, Vondra K, Pelikánová T, Vlcek P, Hill M, Zamrazil V (2005). Insulin sensitivity and counter-regulatory hormones in hypothyroidism and during thyroid hormone replacement therapy. *Clinical Chemistry and Laboratory Medicine*.

[74] Handisurya A, Pacini G, Tura A, Gessl A, Kautzky-Willer A (2008). Effects of thyroxine replacement therapy on glucose metabolism in subjects with subclinical and overt hypothyroidism. *Clinical Endocrinology*.

[75] Dimitriadis G, Mitrou P, Lambadiari V (2006). Insulin action in adipose tissue and muscle in hypothyroidism. *Journal of Clinical Endocrinology & Metabolism*.

[76] Brenta G, Celi FS, Pisarev M, Schnitman M, Sinay I, Arias P (2009). Acute thyroid hormone withdrawal in athyreotic patients results in a state of insulin resistance. *Thyroid*.

[77] Owecki M, Nikisch E, Sowiński J (2006). Hypothyroidism has no impact on insulin sensitivity assessed with HOMA-IR in totally thyroidectomized patients. *Acta Clinica Belgica*.

[78] Harris PE, Walker M, Clark F, Home PD, Alberti KGMM (1993). Forearm muscle metabolism in primary hypothyroidism. *European Journal of Clinical Investigation*.

[79] Dessein PH, Joffe BI, Stanwix AE (2004). Subclinical hypothyroidism is associated with insulin resistance in rheumatoid arthritis. *Thyroid*.

[80] Tuzcu A, Bahceci M, Gokalp D, Tuzun Y, Gunes K (2005). Subclinical hypothyroidism may be associated with elevated high-sensitive C-reactive protein (low grade inflammation) and fasting hyperinsulinemia. *Endocrine Journal*.

[81] Al Sayed A, Al Ali N, Bo Abbas Y, Alfadhli E (2006). Subclinical hypothyroidism is associated with early insulin resistance in Kuwaiti women. *Endocrine Journal*.

[82] Brenta G, Berg G, Arias P (2007). Lipoprotein alterations, hepatic lipase activity, and insulin sensitivity in subclinical hypothyroidism: response to L-T4 treatment. *Thyroid*.

